# The Edinburgh Social Cognition Test (ESCoT): Examining the effects of age on a new measure of theory of mind and social norm understanding

**DOI:** 10.1371/journal.pone.0195818

**Published:** 2018-04-17

**Authors:** R. Asaad Baksh, Sharon Abrahams, Bonnie Auyeung, Sarah E. MacPherson

**Affiliations:** 1 Department of Psychology, School of Philosophy, Psychology, and Language Sciences, University of Edinburgh, Edinburgh, United Kingdom; 2 Centre for Cognitive Ageing and Cognitive Epidemiology (CCACE), University of Edinburgh, Edinburgh, United Kingdom; 3 Autism Research Centre, Department of Psychiatry, University of Cambridge, Cambridge, United Kingdom; Radboud Universiteit, NETHERLANDS

## Abstract

Current measures of social cognition have shown inconsistent findings regarding the effects of healthy aging. Moreover, no tests are currently available that allow clinicians and researchers to examine cognitive and affective theory of mind (ToM) and understanding of social norms within the same test. To address these limitations, we present the Edinburgh Social Cognition Test (ESCoT) which assesses cognitive and affective ToM and inter- and intrapersonal understanding of social norms. We examined the effects of age, measures of intelligence and the Broader Autism Phenotype (BAP) on the ESCoT and established tests of social cognition. Additionally, we investigated the convergent validity of the ESCoT based on traditional social cognition measures. The ESCoT was administered alongside Reading the Mind in Films (RMF), Reading the Mind in Eyes (RME), Judgement of Preference and Social Norm Questionnaire to 91 participants (30 aged 18–35 years, 30 aged 45–60 years and 31 aged 65–85 years). Poorer performance on the cognitive and affective ToM ESCoT subtests were predicted by increasing age. The affective ToM ESCoT subtest and RMF were predicted by gender, where being female predicted better performance. Unlike the ESCoT, better performance on the RMF was predicted by higher verbal comprehension and perceptual reasoning abilities, while better performance on the RME was predicted by higher verbal comprehension scores. Lower scores on inter-and intrapersonal understanding of social norms were both predicted by the presence of more autism-like traits while poorer interpersonal understanding of social norms performance was predicted by increasing age. These findings show that the ESCoT is a useful measure of social cognition and, unlike established tests of social cognition, performance is not predicted by measures of verbal comprehension and perceptual reasoning. This is particularly valuable to obtain an accurate assessment of the influence of age on our social cognitive abilities.

## Introduction

The study of social cognition is concerned with the higher-order cognitive processes that allow individuals to interpret the behaviors of others [1]. These abilities allow us to process and understand social information in order to respond appropriately in everyday interactions [2–5]. Social cognition includes abilities such as theory of mind (ToM; i.e., the ability to recognise other people’s mental states to understand and predict their behavior), emotion recognition, empathy, moral judgments and the understanding of social norms [6, 7].

Healthy aging is associated with reliable improvement in emotional well-being [8] and social functioning [9]. Although social network size decreases with age, older adults’ social interactions with individuals who remain within their social networks are rated as being more satisfying [10]. Life experience is thought to influence how people process and respond to social information (e.g., [11]). For example, older adults are thought to be more receptive to emotional cues when making social judgements compared to younger adults (see [12]). Yet, some studies examining individuals’ ability to understand and evaluate relevant social information have reported poorer performance in healthy older adults compared to younger adults [13, 14].

One of the most extensively studied aspects of social cognition in healthy aging is ToM [14]. More recently, it has been argued that ToM is not a one-dimensional concept, but processes differ based on whether they refer to cognitive or affective judgements [15]. Cognitive ToM is defined as the ability to make inferences about the thoughts, intentions and beliefs of another individual. Affective ToM refers to the ability to make inferences about what another individual is feeling [15–17]. Age-related differences have been found where older adults perform more poorly compared to their younger counterparts on tests such as Reading the Mind in the Eyes (RME) [18], Faux Pas stories (a verbal story based test that requires participants to make ToM inferences from short interactions involving social norms violations and non-social norm violations) [19] and Happé’s Strange Stories, among others [20–29]. Yet, other studies have found age-related improvements in favour of older adults such as Happé *et al*. [29] or equivalent performance between younger and older adults [25, 30–35]. Potentially, age-related differences may be related to one aspect of ToM but not the other, for example cognitive ToM but not affective ToM. However, research into possible dissociations between cognitive and affective ToM has yielded mixed findings. In perspective taking tests which assess cognitive ToM, older adults perform more poorly than younger adults [20, 22, 23, 26, 30, 36–38]. Nonetheless, other authors have failed to find age-related differences in cognitive ToM [30, 31]. Affective ToM has been examined using tests such as the RME [18] where individuals are required to make inferences from the eye region of photographs. Older adults have been found to perform significantly more poorly than younger adults [20, 21, 36, 39, 40]. Video based ToM tests have also shown that older adults perform significantly more poorly than younger adults [28, 40]. However, Castelli *et al*. [30] and Li *et al*. [32] have both reported comparable performance between younger and older adults on the RME. Moreover, story-based affective ToM tests such as the Faux Pas test [19] have less consistently reported age-related differences with some studies reporting poorer performance with age [35] but others not reporting age-related differences [33]. Overall, it is unclear how social cognitive abilities, specifically cognitive and affective ToM abilities are affected by aging when the performance of older adults is compared to younger adults. A possible reason for the inconsistencies in the literature could be related to the way in which researchers assess ToM [13].

The aging literature has tended to assess the influence of age on the distinct components of ToM using different tests (e.g., [36]), and these paradigms vary in both their stimuli type and level of difficulty. For example, affective ToM has been examined using tests involving visual-static stimuli such as Tom’s taste test [23] and the RME [39]. In contrast, cognitive ToM has been examined using verbal vignettes [41] and visual-dynamic false belief story tests [20]. Existing tests of social cognition have been criticised as they require participants to read factual or fictional information regarding multiple characters and process mental state information. Poor performance could be a secondary consequence of broader cognitive difficulties [42]. Moreover, few aging studies have compared affective and cognitive ToM within the same test, making it difficult to contrast the influence of age on tests that are not directly comparable. Recently, Bottiroli *et al*. [22] attempted to measure cognitive and affective ToM using the Faux Pas test. They demonstrated that compared to younger adults, older adults performed poorer on cognitive ToM, but showed intact affective ToM abilities. Yet, some authors have argued that the Faux Pas imposes demands on both cognitive and affective ToM [13]. This test was designed before researchers explicitly regarded ToM as a multidimensional process and so there is no clear distinction between cognitive and affective ToM. Moreover, we would argue that the Faux Pas is a measure of affective ToM, as well as social norm understanding, since it primarily requires the participant to understand that a protagonist’s feelings have been hurt by a social norm violation.

One important aspect of social cognition which has not typically been assessed in the aging literature is the ability to understand social norms from interpersonal and intrapersonal perspectives. While intrapersonal understanding of social norms has been explored in studies of dementia [43], adults with Autism Spectrum Disorders (ASD) [7] and patients with schizophrenia and bipolar disorder [6], few studies have examined this ability in healthy aging. In one of the only studies exploring interpersonal understanding of social norms in healthy aging, Halberstadt, Ruffman, Murray, Taumoepeau and Ryan [44] found that older adults were poorer at discriminating between socially appropriate and inappropriate behaviours from short videos of social interactions compared to younger adults.

Performance on social cognition tests have been shown to be influenced by variables such as personality traits and measures of IQ (e.g., verbal comprehension and perceptual reasoning). Charlton *et al*. [45] has argued that age-related difficulties in ToM are not independent of measures of intelligence. They found that the association between age and ToM abilities as measured by Happé’s Strange Stories test was fully mediated by perceptual reasoning and partially mediated by verbal comprehension. Further studies have found correlations between ToM and verbal abilities [24] and have shown that perceptual reasoning performance accounts for age-related differences, again on Happé’s Strange Stories test [28]. These findings suggest that some tests may not be simply assessing our social cognitive abilities and this has important implications for interpretations of age-related differences in performance.

A hallmark characteristic of ASD is pronounced impairments in social cognition [46]. Moreover, research has shown that difficulties in social cognition are responsible for social functioning impairment in ASD [47], suggesting that social cognitive abilities are important contributions to the quality of an individual’s social interactions. This finding is relevant for the present study since characteristics typically found in adults with ASD are continuously distributed within the general population [48–50]. Indeed, subclinical autistic-like traits referred to as the Broad Autism Phenotype (BAP) [51] within the general population are related to reductions in social cognitive ability [48]. Individuals who exhibit more BAP traits report experiencing more social and interpersonal problems [50, 52]. Additionally, recent evidence suggests that BAP traits in older adults are associated with lower levels of social support, and increased self-reported levels of depression and anxiety [53]. Given the findings discussed above, it would be of interest to examine the relationship between the ESCoT, measures of intelligence and the BAP, and compare these to the findings of established tests.

To our knowledge, no tests are currently available in the literature that allow clinicians and researchers to examine different aspects of social cognition such as cognitive and affective ToM and understanding of social norms within the same test. Yet, some authors have argued that reliable assessments of a given construct should have multiple measures and these should differ in modality [54]. This could possibly be the reason for contradictory findings in the aging literature [13]. Moreover, while tests like the Movie for the Assessment of Social Cognition (MASC) [55], the Awareness of Social Inference Test (TASIT) [56], the Awkward Moments Test [57] and the Empathic Accuracy Paradigm [58] already exist and are all useful indices of social cognitive functioning, they are not without their limitations. For instance, the TASIT [56] uses excerpts from short interactions so lacks important contextual information, the MASC [55] is dubbed in English, the Awkward Moments Test [57] uses television adverts of exaggerated interactions and the Empathic Accuracy Paradigm [58] uses scenes from hidden filming which limits the range of mental states to be inferred.

We attempted to address these issues using a novel test of social cognition called the Edinburgh Social Cognition Test (ESCoT). We devised the ESCoT to explicitly measure both cognitive and affective ToM in the same test. The ESCoT also provides a much-needed measure of interpersonal and intrapersonal social norm understanding. Few tests measure more than one social cognitive ability in a single test, but the ESCoT provides four distinct and potentially informative insights into social cognitive abilities.

The aims of this study were to investigate the relationship between the ESCoT and a) age, b) measures of intelligence and c) the BAP in comparison to established tests. Additionally, we sought to examine convergent validity between the ESCoT and other measures of social cognition. By closely examining different social cognitive abilities in a systemic manner using the ESCoT, this study sought to shed new light on the consequences of aging on social cognitive abilities in younger, middle-aged and older adults.

## Methods

### Participants

A total of 91 healthy participants were recruited for this study: 30 aged between 18 and 35 years (15 male, 15 female), 30 aged between 45 and 60 years (15 male, 15 female) and 31 aged between 65 and 85 years (14 male, 17 female). The participants’ demographic information is reported in Table 1. None of the participants had any self-reported history of neurological or psychiatric disorders based on the Wechsler Adult Intelligence Scale (WAIS-III) exclusion criteria [59]. Participants were recruited from online advertisement, through a Psychology Department volunteer panel, and were reimbursed for their time. Written informed consent to participate in the study was obtained from each participant. The study was approved by the School of Philosophy, Psychology and Language Sciences (Psychology) Ethics committee at the University of Edinburgh.

**Table 1:** Summary of demographic information.

	Age groups				
	Younger adults *N* = 30	Middle-aged adults *N* = 30	Older adults *N* = 31	Sig*	ηp^2^ (*d*)
Age (*SD*)	26.20 (5.21)	50.60 (5.77)	72.45 (6.05)	-	-
Males:Females	15:15	15:15	14:17	-	-
Years of full-time education	17.03 (2.82)	15.53 (2.86)	14.58 (2.88)	O<Y	.12 (.74)

Y = Younger adults; M = Middle-aged adults, O = Older adults.

*Analyses were conducted using one-way ANOVAs, post hoc testing were conducted using Gabriel’s procedure for multiple comparisons. All *p* < .05.

### Measures

The Wechsler Abbreviated Scale of Intelligence, Second Edition (WASI-II) [60] was administered as a measure of verbal comprehension and perceptual reasoning. Participants completed four subtests: Vocabulary; Similarities; Block Design; and Matrix Reasoning. Scores from each of the four subtests were converted to age-adjusted standardised scores. The Vocabulary and Similarities subsets provided a Verbal Comprehension Index (VCI) and Block Design and Matrix Reasoning provide a Perceptual Reasoning Index (PRI) [60, 61].

The Autism Quotient (AQ) [62] was administered to assess traits related to the autism spectrum. The Empathy Quotient (EQ) [63] was administered to measure the ability to identify and understand the thoughts and feelings of others and to respond to these with appropriate emotions. The Systemizing Quotient assessed the drive to analyse or construct systems such as mechanical systems. All questionnaires were self-report and participants completed them electronically. For the AQ (maximum score = 50), the higher the score, the more autistic-like characteristics the individual possessed. For the EQ (maximum score = 80), higher scores suggested higher levels of empathy. For the SQ (maximum score = 150), higher scores suggested stronger interest systems, for example the drive to construct systems or to understand the underlying rules that govern a system.

#### The Edinburgh Social Cognition Test (ESCoT)

The Edinburgh Social Cognition Test (ESCoT) measured four social cognitive abilities: cognitive ToM; affective ToM; interpersonal understanding of social norms and intrapersonal understanding of social norms.

The ESCoT consisted of 11 dynamic, cartoon-style social interactions (each approximately 30 seconds long): 1 practice interaction, 5 interactions involved a social norm violation and 5 portrayed everyday interactions that did not involve social norm violations. Each animation had a different context and specific questions relating to that context. The animation was presented in the middle of a computer screen and at the end of each animation, a static storyboard depicting a summarised version of the interaction was presented (see S1 Fig in the supplementary materials). The storyboard remained on the screen for the duration of the trial. Participants were asked to describe what had occurred in the interaction. Then participants were asked one question to assess each of the four subtests of social cognition. To allow participants to give their optimal interpretation of each interaction and capture the quality of their response, they were prompted if they gave a limited response or their response lacked important information from the interaction. They were prompted with the question, ‘Can you tell me more about what you mean by that?’ or ‘Can you explain that in a little bit more detail?’. Each participant was prompted only once for each question.

Each response was scored based on the quality of the answer with maximum points awarded for responses that successfully extracted and integrated the relevant information from the interaction and articulated this response in a contextually specific manner. Importantly, response length was not related to quality; participants could score maximum points with a minimal response. For scoring of the intrapersonal understanding of social norms subtest, responses that considered the social nuances of the interaction were scored more highly than responses that highlighted personal attributes of the participant. Each question was awarded a maximum of 3 points, resulting in a score of 12 points for each social interaction. The total maximum score for the test was 120 points. The ESCoT took appropriately 20–25 minutes to complete and the animations were viewed on VLC media player. Researchers interested in using the ESCoT can contact the corresponding author to obtain the full test with scoring instructions.

#### Reading the Mind in the Eyes (RME) [18]

The RME was administered to assess affective ToM. Participants were presented with photographs of the ocular region of different human faces and were required to make a force-choice response from four adjectives (one target and three foils) which best described what the individual was thinking or feeling. If participants were unsure or unfamiliar with an adjective, they were provided with a glossary of the adjectives and their definitions to clarify what each word meant. Participants kept this glossary throughout testing and could refer to it when required. Responses were recorded verbally and 1 point was awarded for each correct answer, giving a total score of 36.

#### Reading the Mind in Films (RMF) [64]

The RMF was administered to assess affective ToM. Participants viewed short scenes from feature films and were instructed to make a forced-choice response from four adjectives (one target and three foils) that best described what the protagonist was thinking or feeling at the end of the scene. Similar to the RME, participants were provided with a glossary of the adjectives for clarification and responded verbally. A correct response was awarded 1 point, giving a total score of 22.

#### Judgement of Preference (JoP) [65]

The JoP assessed a participant’s ability to make affective ToM judgements of a character while inhibiting their own preferences. This version consisted of a pre-experimental condition and two experimental conditions, each comprising of twelve trials each. In the pre-experimental condition, participants were instructed to choose the item that they liked the most out of 4 items. Following this, participants were presented with a small circular face in the middle of a computer screen with 4 objects in the four corners. In the affective condition, participants were told to choose the item the face in the middle of the screen liked. In the physical condition, participants were asked to identify the item that the face was looking at. Participants touched the item in the correct position on the screen of a touch-screen computer. Each participant was instructed to respond as quickly but as accurately as possible. The affective and physical conditions were counterbalanced. A correct response was given 1 point with a maximum score of 12 per condition.

#### Social Norms Questionnaire (SNQ) [66]

The SNQ examined intrapersonal understanding of social norms. It was originally developed to screen patients for potential behaviour changes and is administered to examine how well participants understand the social standards that govern their behaviour in mainstream culture. Participants were given a list of behaviours (e.g., tell a stranger you don’t like their hairstyle?) and asked to indicate whether or not each of the behaviours was socially acceptable to perform in the presence of a stranger or acquaintance, not a close friend or family member. A total score (maximum score = 22) was calculated, with higher scores reflecting better performance.

### Procedure

Participants completed all six tasks in a single session, which took approximately two hours to complete. The order of the tasks was kept the same for each participant.

### Statistical analyses

The effects of age, intelligence (verbal comprehension and perceptual reasoning) and the BAP (AQ, EQ and SQ) on the ESCoT and established tests of social cognition were investigated using hierarchical multiple regression analysis. In the first stage, the background predictors (age, gender, years of education, measures of IQ) which showed a correlation with the outcome variables (subtests of the ESCoT, ESCoT total scores and established social cognition tests) at a pre-specified significance level of *p*<0.20 was entered into the analysis [67] using the enter method. While some researchers have suggested that all relevant variables should be included in the regression model regardless of their significance, this approach can result in numerically unstable estimates and large standard errors [68]. We chose a significance level of *p*<0.20 over more traditional levels such as *p*<0.05 because *p*<0.05 can fail in identifying variables known to be important, and simulation studies have shown that a cut-off of *p*<0.20 yields better outcomes than a cut-off of *p*<0.05 [68, 69]. The scores of VCI and PRI were entered into the first stage independently along with the other background predictor variables in separate regression models. In the second stage, AQ, EQ and SQ scores were entered using the stepwise method (entry criterion *p*<0.05, removal criterion *p*>0.10) to examine their effect on performance. Furthermore, adjusted scores based on the regression analyses were calculated. These age adjustments were calculated using the unstandardized β coefficients from the regression analysis and the mean age of the sample, the calculations for these can be found in the supplementary materials (S2 Fig). To investigate the relationship between the ESCoT and standard tests of social cognition, correlational analyses were conducted to validate the ESCoT against established tests.

## Results

Table 2 demonstrates the preliminary correlational analyses between cognitive ToM, affective ToM, inter- and intrapersonal understanding of social norms with VCI scores, PRI scores, age, years of education and gender. Variables with correlations that were significant at the *p*<0.20 level were included in the regression analysis. Table 3 shows a summary of the regression analyses for the subtests of the ESCoT.

**Table 2:** Correlational analysis between the background predictors and measures of the ESCoT.

Outcome variable	Age	Years of education	Gender	VCI	PRI
Cognitive ToM	–0.32*	0.18*	0.01	0.12	0.04
Affective ToM	–0.17*	0.09	0.23*	0.15*	0.18*
Interpersonal understanding of social norms	–0.38*	0.12	0.06	–0.08	–0.09
Intrapersonal understanding of social norms	–0.16*	–0.09	–0.13	–0.09	–0.11

*p<0.20.

Predictor variables which correlated with the outcome variable at the *p*<0.20 level met criteria for inclusion in the regression model. Predictor variables with correlations p>0.20 did not meet criteria for inclusion in the regression model. Results of the regression analyses that included the correlated variables can be seen in Table 3. VCI = Verbal Comprehension Index; PRI = Perceptual Reasoning Index.

**Table 3:** Regression analyses for the subtests of the ESCoT with VCI and PRI scores.

	Model 1 summary	Significant predictors in Model 1	Excluded predictors in Model 2	F–change & ΔR^2^	Significant predictors in Model 2
Cognitive ToM	R^**2**^ = 0.11, *p*<0.01	Age (*p*<0.01)	AQ, EQ & SQ	–	–
Interpersonal Understanding of Social Norms	R^**2**^ = 0.14, *p*<0.01	Age (*p*<0.001)	EQ & SQ	F–change = 10.55, *p*<0.01, ΔR^**2**^ = 0.09	Age (*p*<0.001) & AQ (*p*<0.01)
Intrapersonal Understanding of Social Norms	R^**2**^ = 0.03, *p*>0.05	–	EQ & SQ	F–change = 7.27, *p*<0.01, ΔR^**2**^ = 0.08	AQ (*p*<0.01)
**VCI**: Affective ToM	R^**2**^ = 0.13, *p*<0.05	Age (*p*<0.05) & Gender (*p*<0.05)	AQ, EQ & SQ	–	–
**PRI**: Affective ToM	R^**2**^ = 0.12, *p*<0.05	Age (*p*<0.05) & Gender (*p*<0.05)	AQ, EQ & SQ	–	–

**ESCoT total scores and IQ scores.** The ESCoT total scores correlated with age (r = –0.42, *p*<0.20). Years of education (r = 0.13, *p*>0.20), gender (males = 1, females = 2, r = 0.09, *p*>0.20), VCI scores (r = 0.11, *p*>0.20) and PRI scores (r = 0.09, *p*>0.20) did not correlate with ESCoT total scores at *p*<0.20. Therefore, these variables did not meet criteria for inclusion in the model.

In the regression model, age was the only significant predictor of ESCoT performance (*p*<0.001, R^**2**^ = 0.18). The inclusion of AQ, EQ and SQ scores produced a significant F-change (F-change = 5.44, *p*<0.05, ΔR^**2**^ = 0.05). In the final model, only age (*p*<0.001) and AQ scores (*p*<0.05) were significant predictors of ESCoT performance, with older age and higher AQ scores predicting poorer performance on the ESCoT. EQ and SQ scores were excluded as predictors from the final regression model.

**RME and IQ scores.** For the RME scores, VCI scores (r = 0.28, *p*<0.20) met criteria for inclusion in the first stage of the analysis. Age (r = –0.10, *p*>0.20), years of education (r = –.07, *p*>0.20) and gender (r = –0.06, *p*>0.20) were not included. In the first model, VCI scores (*p*<0.05, R^**2**^ = 0.06) predicted performance on the RME. Higher VCI scores predicted better RME performance. AQ, EQ and SQ scores were not retained in the final model.

**RMF and IQ scores.** Gender (r = 0.23, *p*<0.20), VCI scores (r = 0.38, *p*<0.20) and PRI scores (r = 0.27, p<0.20) met the criteria for inclusion in the regression models. Age (r = .02, *p*>.20) and years of education (r = –0.03, *p*>0.20) did not correlate with RMF scores at *p*<0.20. In the first model, VCI scores (*p*<0.01) and gender (*p*<0.05) were significant predictors of RMF performance (*p*<0.001, R^**2**^ = 0.18). Female participants and participants who had higher VCI scores performed better on the RMF. No variables were entered into the model in the second stage as they did not meet criteria for inclusion.

In a regression model with PRI scores, both gender (*p*<0.05) and PRI scores (p<0.05) were predictors of RMF scores (*p*<0.01, R^**2**^ = 0.13). Female participants and participants with higher PRI scores were associated with better performance on the RMF. AQ, EQ and SQ scores were not retained in the final model.

**SNQ and IQ scores.** Age (r = 0.14, *p*<0.20) and VCI scores (r = 0.23, *p*<0.20) met criteria for inclusion. Gender (r = 0.07, *p*>0.20), years of education (r = –0.12, *p*<0.20) and PRI scores (r = 0.06, *p*>0.20) did not meet criteria for inclusion in the regressions. However, all regression analyses were not significant (all *p*>0.10).

**JoP and IQ scores.** Age (r = 0.05, *p*>0.20), years of education (r = –0.05, *p>*0.20) gender (r = –0.13, *p*>0.20), VCI scores (r = –0.06, *p>*0.20) and PRI scores (r = –0.09, *p*>0.20) did not meet criteria for inclusion in the regression models. Moreover, all regression analyses for the JoP were not significant (all *p*>0.10).

### Age adjusted scores

#### Cognitive ToM

The regression analysis demonstrated a negative association with age; as age increased, performance on cognitive ToM decreased. Rather than producing separate normative data for each age group, we suggest that raw cognitive ToM scores should be adjusted for age accordingly: 18–22 years old = –1 point, 23–77 years old = no change in raw score and 78 years and older = +1 point.

#### Affective ToM

The regression analysis revealed that age negatively predicted performance on affective ToM. As participants’ ages increased, performance on affective ToM decreased. Therefore, raw affective ToM scores should be adjusted for age: 18–26 years old = –1 point, 27–73 years old = no change in raw score and 74 years and older = +1 point.

Gender predicted performance on affective ToM with being female predicting better performance better than being male. However, the difference between the male and female groups was only 0.36 standard deviations (less than 1 point on the ESCoT). Therefore, it is not necessary to adjust the raw affective ToM scores for gender.

#### Interpersonal understanding of social norms

Since the regression analysis revealed that age predicted performance on interpersonal understanding of social norms, raw scores should be adjusted as follows: 18–19 years old = –2 points, 20–34 years old = –1 point, 35–65 years old = no change, 66–80 years old = +1 point and 81 years and older = +2 points.

### Correlations between the ESCoT and established tests

Correlational analyses with the Holm correction for multiple comparisons showed that the ESCoT significantly correlated with the RME (r = 0.33, *p*<0.01) and showed a trend towards significance with the SNQ (r = 0.19, *p*>0.05). The RME correlated with the RMF (r = 0.38, *p*<0.001) and SNQ (r = 0.34, *p*<0.01). The RMF also showed a trend towards significance with the SNQ (r = 0.19, *p*>0.05). None of the tests significantly correlated with the JoP (all *p*>0.10).

### ESCoT inter-rater reliability and internal consistency

To establish the reliability of the scoring, we calculated inter-rater reliability for the ESCoT using intraclass correlation (ICCs). A second independent rater scored a sample of 5 participants from each age group. The consistency (ICCs) for the 15 ratings was 0.90, indicating high inter-rater reliability.

We assessed internal consistency for the ESCoT by calculating Guttman's Lambda 4 reliability coefficient. This method has been shown to be a better measure of internal consistency than Cronbach’s alpha [70]. Guttman's Lambda 4 reliability coefficient for the ESCoT was 0.70 which is acceptable [71].

## Discussion

The current study presented a new within subjects’ measure of social cognition that assesses cognitive and affective ToM, as well as intra- and interpersonal social norm understanding, within the same test. We examined the effects of age, measures of intelligence and the BAP on the ESCoT and established tests of social cognition. Additionally, we investigated the relationship between the ESCoT and established measures of social cognition. Total ESCoT scores were predicted by the age of participants and their AQ scores, here increasing age and AQ scores resulted in poorer performance. Investigation of the subcomponents of the ESCoT revealed that performance on cognitive ToM was significantly predicted by age, with increasing age resulting in decreased performance on cognitive ToM. Affective ToM was also predicted by age but also gender; in this instance, better performance was associated with being younger and female. Moreover, performance on interpersonal understanding of social norms was predicted by age and AQ scores–increasing age and AQ scores were predictive of poorer performance. On the subtest of intrapersonal understanding of social norms, higher AQ scores predicted poorer performance.

Notably, the ESCoT total score and sub-test measures were not associated with the two measures of intelligence; verbal comprehension (VCI) and perceptual reasoning (PRI). This contrasts with performance on some of the more standard tests of social cognition. In the present study, we found that participants with higher verbal comprehension scores performed better on the RME, while RMF performance was significantly predicted by measures of verbal comprehension, perceptual reasoning and gender. Here, female participants and those with higher verbal comprehension and perceptual reasoning scores performed better on this measure of affective ToM. The correlation analysis demonstrated that ESCoT total scores significantly correlated with the RME and showed a trend towards significance with the SNQ, indicating convergent validity.

Similar to previous findings in the literature which have demonstrated aged-related difference in cognitive ToM [20, 22, 23, 26, 30, 36–38], age predicted poorer performance in cognitive ToM on the ESCoT. This provides further evidence that, as we get older, we experience difficulties in our ability to infer what another individual is thinking. Moreover, we found that increasing age predicted poorer performance in participants’ ability to infer what another is feeling, comparable to some [21, 28, 36, 40], but in contrast to other studies [22, 33, 35]. It could be argued that the findings here are more representative of the population, as we included adults aged 18–85 years while Bottiroli *et al*. [22] only included younger and older adults. Or, as Henry *et al*. [13] have argued, age-related differences can be the consequence of the type of task used. For example, Phillips *et al*. [34] examined how well older adults were able to assess the severity of contextual emotions of individuals in short stories. They found younger and older adults did not significantly differ in this ability. However, forced choice tests offer limited insights in understanding the relationship between age and social cognition. Primarily because there are few real-world social interactions where inferring what another person is feeling is forced-choice in nature. Overall, these results suggest that the process of healthy aging is associated with difficulties in both components of ToM.

To our knowledge, this is the first study to assess the ability to understand social rules in the same test as ToM and explicitly examine interpersonal (did X behave as other people should behave?) and intrapersonal (would you have acted the same as X?) understanding of social norms. Age was found to predict poorer performance on interpersonal understanding of social norms. These findings add to the preliminary findings of Halberstadt *et al*. [44] who showed poorer performance of older adults compared to younger adults on interpersonal understanding of social norms. We provide a novel finding in regards to intrapersonal understanding of social norms; we showed that the age of participants was not a predictive variable of performance. This suggests that age does not impact our own knowledge of how we should behave in social situations, and not all our social cognitive abilities are negatively affected by age. Both of these findings demonstrate that understanding of social norms warrants further investigation.

Although both cognitive and affective ToM were affected negatively by age, we do provide some evidence for a dissociation between the two processes in that performance is predicted by different demographic variables. This is analogous to the findings that cognitive and affective ToM correlate with different cognitive processes [22]. Cognitive ToM performance was negatively predicted by age while affective ToM was significantly predicted by age and gender. Like Duval *et al*. [23], we found that both cognitive and affective ToM show impairments with advancing age in the same study. The advantage in this study was that we were able to measure cognitive and affective ToM within the same test, unlike Duval *et al*. [23] who relied on different tests to measure these abilities and was therefore unable to control for task difficulty. Gender was only found to predict performance on affective ToM; this is similar to research found in the literature which has shown that women are significantly better at inferring what a character is feeling compared to men [18, 72, 73]. These results show that, as well as considering the consequences of aging on our social cognitive abilities, we should consider the gender of the sample population. Furthermore, they highlight the importance of adopting social cognitive tests that assess cognitive and affective ToM separately and suggest composite tests are not appropriate to accurately examine ToM in aging populations. Using within subjects tests are essential if we are to better understand whether aging does indeed affect cognitive and affective ToM in the same way.

The only test of social cognition that was associated with the measures of the BAP was the ESCoT, suggesting that perhaps the ESCoT is more sensitive to difficulties in social abilities of individuals on the BAP compared to established tests. Here, lower scores in inter- and intrapersonal understanding of social norms were associated with higher scores on the AQ. Additionally, we found that the presence of more autistic traits predicted poorer overall performance on the ESCoT. These are novel findings but makes sense in the context of the BAP, as impaired social cognition is related to the milder social-behavioral phenotype described as part of the BAP [48, 50, 52]. Research on the understanding of social norms in healthy aging is limited but these findings are in line with research that show that adults with ASD perform poorer than controls on tests such as the Faux Pas which implicitly assess social norms understanding [74]. However, the relationship between ASD and intrapersonal understanding of social norms is less clear and requires further investigation For example, Baez *et al*. [7] found that adults with ASD do not significantly differ on this ability compared to controls. Nonetheless, the observed relationship between the ESCoT and the AQ demonstrates that this new test of social cognition may offer new insights into the relationship between the BAP and social cognition in healthy aging populations and may be valuable in ASD research.

An advantage of the ESCoT over other tests of social cognition, is that overall performance was not related to measures of IQ, namely verbal comprehension and perceptual reasoning performance. However, this is not the typical finding with social cognition measures. Charlton *et al*. [45] found that performance on Happé’s Strange Stories test (a composite ToM task) was fully mediated by performance IQ, executive function, and information processing speed and was partially mediated by verbal IQ. Moreover, again on Happé’s Strange Stories test, Sullivan and Ruffman [28] both found that ToM abilities were related to perceptual reasoning abilities. In both the current study and previous studies in the literature [64, 72, 75], performance on the RME and RMF was found to be predicted by verbal comprehension. In one study, the only significant predictor of performance on the RME test was verbal comprehension which accounted for 11.7% of the variance [76]. This has implications for studies using the RME and RMF to investigate affective ToM as they appear to be tests of verbal comprehension as well as affective ToM.

A limitation of the present study is that we did not examine the relationship between executive functions and the ESCoT. Given that social cognition has been associated with executive abilities in aging [22, 23, 27, 72], future work might explore potential associations between the ESCoT and processes such as inhibition, set-shifting and updating. Finally, it has been suggested that the clinical assessment of social cognition should emulate the way in which individuals process social situations in everyday life [4]. As argued by Henry *et al*. [13], dynamic-visual information such as images depicting a social interaction that lead to a protagonist in a particular mental state is more ecologically valid and information-rich compared to verbal narratives. Consequently, dynamic cartoons were chosen as the mode of presentation in the ESCoT. This allowed perceivers to use many more cues to make inferences [77], similar to real-life. Videos of real individuals interacting would be the ideal stimuli for assessing social cognitive abilities to maximise ecological validity. However, social interactions are highly complex [78] and social information can be difficult to control in real interactions. Therefore, it may be difficult to separate the specific social cognitive process that the test is intending to measure. With animated characters, specific social cognitive abilities can be more easily isolated and individual social differences can be controlled; essentially all of the parameters can be regulated. For these reasons, we chose to use animated interactions for the ESCoT.

This study is the first to assess cognitive ToM and affective ToM, as well as interpersonal and intrapersonal understanding of social norms within the same test in younger, middle-aged and older adults. We have provided further evidence for similar but distinct components of ToM and evidence for social norm understanding. These findings are useful in furthering our understanding of the consequences of aging on our social cognitive abilities. They also demonstrate specific advantages of the ESCoT over other tests of social cognition. The ESCoT is able to assess distinct aspects of social cognition within a single task and using a within subjects design, allowing for systematic comparisons of these abilities. This important feature of the ESCoT allows it to be a useful test for researchers examining age-related changes in social cognition, and perhaps as a test for clinicians in populations such as adults with ASD, traumatic brain injury patients or psychiatric and neurological patients with suspect social cognitive impairments. As a consequence of its design, the ESCoT can provide researchers and clinicians with an objective measurement of four important social cognitive abilities that are needed to interact with others. This is particularly useful in clinical settings since the results can be used to personalise interventions or educate caregivers about the difficulties the patient might be experiencing in processing social information and interacting with others. In conclusion, these findings show that the ESCoT is a valuable measure of social cognition and, unlike established and standard tests of social cognition, performance is not predicted by measures of verbal comprehension and perceptual reasoning. This is particularly valuable in order to get an accurate assessment of the influence of age on our social cognitive abilities.

## Supporting information

S1 Fig Example interaction from the ESCoT with questions. General comprehension question: Can you tell me what's happening in this story, starting with the first picture and finishing with the last picture? Cognitive ToM: What is the elderly lady thinking? Affective ToM: How does the elderly lady feel at the end of the animation? Interpersonal Understanding of Social Norms: Did the man in the animation behave as other people should behave? Intrapersonal Understanding of Social Norms: Would you have acted the same as the man in the animation? Reprinted under a CC BY license, with permission from the authors of the paper, original copyright 2018.
(TIF)

S2 Fig Formula and calculations for age-adjusted scores for subtests of the ESCoT. (DOCX)

S1 Dataset Regression analysis. (SAV)

S2 Dataset Inter-rater reliability analysis. (SAV)

S3 Dataset Internal consistency analysis. (SAV)
