# Supplementary figures and images for: The Edinburgh Social Cognition Test (ESCoT): Examining the effects of age on a new measure of theory of mind and social norm understanding
Source: PLoS One. 2018 Apr 17;13(4):e0195818. doi: 10.1371/journal.pone.0195818 (PMC5903589; doi:10.1371/journal.pone.0195818)

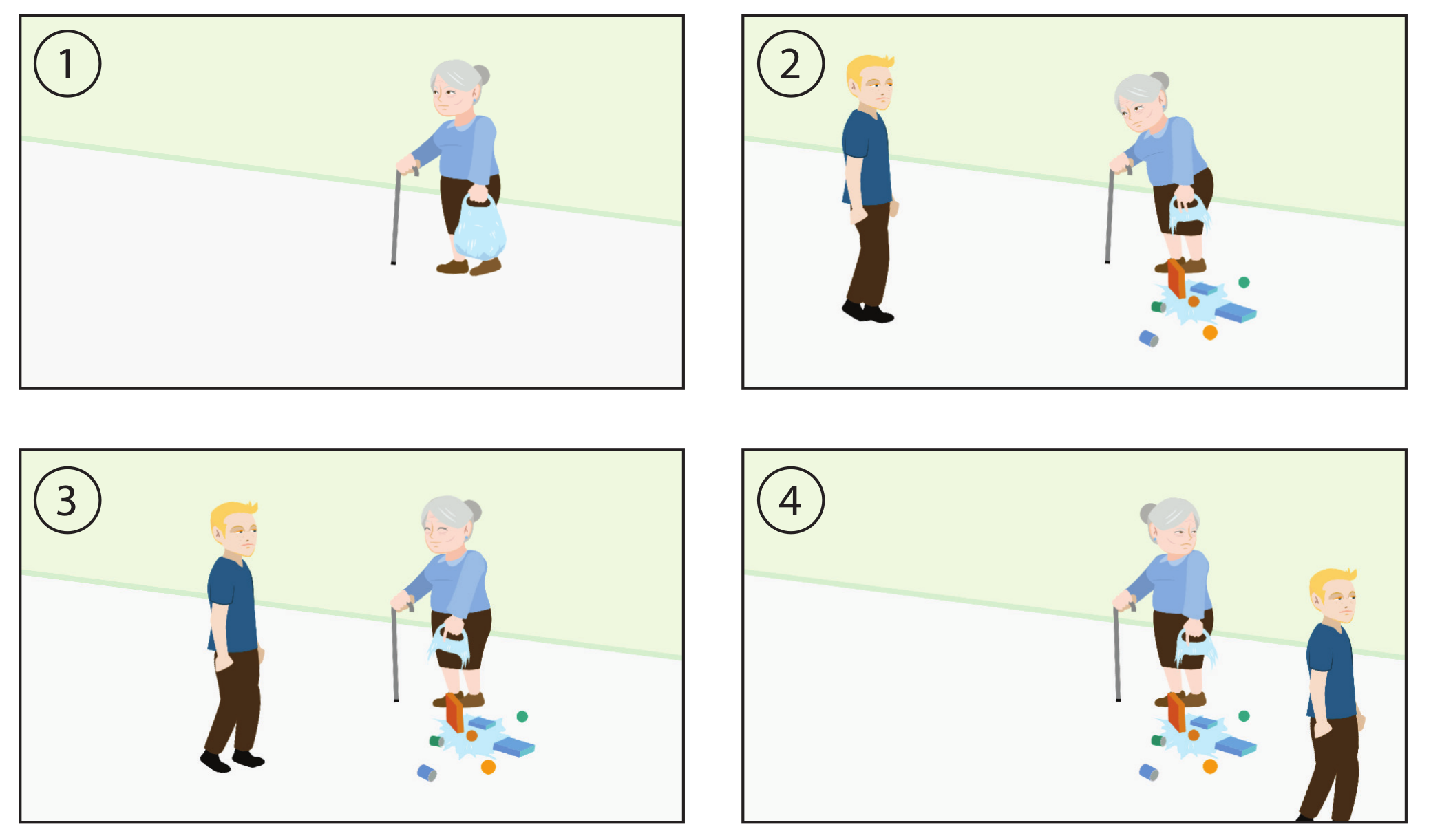

Supplement: S1 Fig — General comprehension question: Can you tell me what's happening in this story, starting with the first picture and finishing with the last picture? Cognitive ToM: What is the elderly lady thinking? Affective ToM: How does the elderly lady feel at the end of the animation? Interpersonal Understanding of Social Norms: Did the man in the animation behave as other people should behave? Intrapersonal Understanding of Social Norms: Would you have acted the same as the man in the animation? Reprinted under a CC BY license, with permission from the authors of the paper, original copyright 2018. (TIF) [file pone.0195818.s001.tif]
