# Supplementary material for: The Edinburgh Social Cognition Test (ESCoT): Examining the effects of age on a new measure of theory of mind and social norm understanding
Source: PLoS One. 2018 Apr 17;13(4):e0195818. doi: 10.1371/journal.pone.0195818 (PMC5903589; doi:10.1371/journal.pone.0195818)
Supplement: S2 Fig — (DOCX) [file pone.0195818.s002.docx]

**S2 Fig. Formula and calculations for age-adjusted scores for subtests of the ESCoT**

General formula:

K = b_1_ (χ – mean (χ))

Cognitive Theory of Mind:

1 = 0.037 (χ – 50)

Affective Theory of Mind

1 = 0.043 (χ – 50)

Interpersonal Understanding of Social Norms:

1 = 0.066 (χ – 50)

To calculate the age adjustments for individuals younger than 20 years old and 81 years old and older:

2 = 0.066 (χ – 50)
